# Fibroblast growth factor 16 stimulates proliferation but blocks differentiation of rat stem Leydig cells during regeneration

**DOI:** 10.1111/jcmm.14157

**Published:** 2019-01-22

**Authors:** Yue Duan, Yiyan Wang, Xiaoheng Li, Jiaying Mo, Xiaoling Guo, Chao Li, Mengyan Tu, Fei Ge, Wenwen Zheng, Jing Lin, Ren‐Shan Ge

**Affiliations:** ^1^ Department of Neonatology, The Second Affiliated Hospital and Yuying Children’s Hospital Wenzhou Medical University Wenzhou Zhejiang China; ^2^ Department of Anesthesiology, The Second Affiliated Hospital and Yuying Children’s Hospital Wenzhou Medical University Wenzhou Zhejiang China; ^3^ Department of Pediatrics Icahn School of Medicine at Mount Sinai New York City New York

**Keywords:** differentiation, FGF16, Leydig cell, proliferation, testosterone

## Abstract

**Objectives:**

We aim to investigate the effects of fibroblast growth factor 16 (FGF16) on Leydig cell regeneration in ethane dimethane sulphonate (EDS)‐treated rat testis.

**Methods:**

We intraperitoneally inject 75 mg/kg EDS to adult male Sprague Dawley rats and then intratesticularly inject FGF16 (0, 10 and 100 ng/testis/day) from post‐EDS day 14 for 14 days. We investigate serum hormone levels, Leydig cell number, gene and protein expression in vivo. We also explore the effects of FGF16 treatment on stem Leydig cell proliferation in vitro.

**Results:**

FGF16 lowers serum testosterone levels (21.6% of the control at a dose of 100 ng/testis) without affecting the levels of luteinizing hormone (LH) and follicle‐stimulating hormone (FSH) on post‐EDS day 28 in vivo. FGF16 increases Leydig cell number at doses of 10 and 100 ng/mg without affecting Sertoli cell number, increases the percentage of PCNA‐positive Leydig cells, and down‐regulates the expression of Leydig cell genes (*Lhcgr*, *Scarb1*, *Star*, *Cyp11a1*, *Cyp17a1* and *Hsd17b3*) and Sertoli cell genes (*Fshr*, *Dhh* and *Sox9*) and their proteins in vivo. FGF16 increases phosphorylation of AKT1 and AKT2 as well as EKR1/2 in vivo, indicating that it possibly acts via AKT1/ATK2 and ERK1/2 pathways. FGF16 also lowers medium testosterone levels and down‐regulates the expression of Leydig cell genes (*Lhcgr*, *Scarb1*, *Star*, *Cyp11a1*, *Cyp17a1* and *Hsd17b3*) but increases EdU incorporation into stem Leydig cells in vitro.

**Conclusions:**

These data suggest that FGF16 stimulates stem and progenitor Leydig cell proliferation but blocks their differentiation, thus lowering testosterone biosynthesis.

## 1 INTRODUCTION

Leydig cells, existing in the interstitium surrounding the seminiferous tubules in the mammalian testis, produce testosterone.^1^, ^2^ Testosterone plays a critical role in stimulating spermatogenesis, maintaining secondary sexual characteristics and promoting muscle bulk and bone health.^3^ Testosterone biosynthesis by Leydig cells relies on pituitary gland‐secreted LH.^4^ Leydig cells possess LH receptor (LHCGR), which is a G‐protein on the cell plasma membrane and responds to LH stimulation.^5^ After its stimulation, extracellular cholesterol is transported inside the Leydig cells via high‐density lipoprotein receptor (SCARB1). Another protein, steroidogenic acute regulatory protein (STAR), is acutely motivated to transport free cholesterol into the inner mitochondrial membrane, where the first steroidogenic enzyme cytochrome P450 cholesterol side‐chain cleavage enzyme (CYP11A1) is present and catalyses the free cholesterol into pregnenolone. Pregnenolone is diffused into the smooth endoplasmic reticulum, where three steroidogenic enzymes, 3β‐hydroxysteroid dehydrogenase (HSD3B1), cytochrome P450 17α‐hydroxylase/C17‐C20 lyase (CYP17A1) and 17β‐hydroxysteroid dehydrogenase 3 (HSD17B3) are present and they catalyse a series of androgen biosynthesis chain reaction of different steroid intermediates from pregnenolone to finally produce testosterone.^6^, ^7^ Besides the acute stimulation of steroidogenesis, LH is a hormone to promote the development of Leydig cells during puberty as the knockout of LHCGR in mice led to the Leydig cell hypoplasia with only the presence of progenitor Leydig cells.^8^, ^9^ However, LH is not required for the initial commitment of stem Leydig cells (the spindle‐shaped cells), as they do not express LHCGR.^10^ Progenitor Leydig cells (the spindle‐shaped cells), which express Leydig cell biomarker LHCGR, CYP11A1, HSD3B1 and CYP17A1,^10^ are still formed in the LHCGR knockout mice.^8^, ^9^ Other factors might be the candidates of the earlier regulation of the stem Leydig cell development.

Leydig cell regeneration can be achieved in adult rat testis after a single intraperitoneal injection of EDS.^11^ During the earlier regeneration, stem Leydig cells highly proliferate and commit into the Leydig cell lineage starting on post‐EDS day 14 by expressing Leydig cell biomarker CYP11A1.^12^, ^13^ On post‐EDS day 28, ovoid immature Leydig cells are differentiated with an expression of a glucocorticoid metabolizing enzyme 11β‐hydroxysteroid dehydrogenase 1 (HSD11B1), a biomarker for Leydig cells at the advanced stages.^12^

By searching the possible growth factors that might regulate Leydig cell development, we re‐analysed the microarray data of transcriptome from EDS‐treated rat testis during the course of Leydig cell regeneration. We found that all 21 fibroblast growth factors (*fgf* isoforms) are present in rat testis with the expression of *Fgf16* being the highest among these factors. Fibroblast growth factors (FGFs) are secreted or anchored proteins that play critical roles in developmental cell processes, including proliferation and differentiation, and exert regulatory, morphological and endocrine and paracrine effects.^14^ FGF16 is a paracrine factor that belongs to a subfamily of FGF9, which includes FGF9, FGF16 and FGF20. The FGF9 subfamily does not possess a classical N‐terminal signal peptide but possesses an internal hydrophobic sequence that functions as a non‐cleaved signal for transporting into the endoplasmic reticulum and secretion from cells.^15^ Interestingly, knockout of FGF9 in mice creates a male‐to‐female sex reversal because of the Leydig cell hypoplasia,^16^ indicating that FGF9 subfamily plays a critical role in Leydig cell development. However, knockout of FGF16 in mice does not have apparent dysfunction of reproduction but a decreased proliferation of heart cells.^17^ Although the level of FGF16 in foetal rodent gonad is low, the abundant expression of FGF16 in adult rat testis indicates that it plays a role in Leydig cell function. In the current study, we used an in vivo EDS‐treated Leydig cell regeneration model and an in vitro stem Leydig cell culture to address the roles of FGF16 in Leydig cell development in the adult testis.

## 2 MATERIALS AND METHODS

### 2.1 Chemicals and kits

FGF16 was purchased from PeproTech (Rocky Hill, NJ). Immulite2000 Total Testosterone kit was purchased from Sinopharm (Hangzhou, Zhejiang, China). Culture medium (M199, DMEM and F12) and Click‐iT EdU (EdU) imaging kit were purchased from Invitrogen (Carlsbad, CA). EDS was purchased from Pterosaur Biotech (Hangzhou, Zhejiang, China). Antibody information was listed in Table S1. Animals were purchased from Shanghai Laboratory Animal Center (Shanghai, China). The use of animals was approved by the Animal Care and Use Committee of Wenzhou Medical University.

### 2.2 Re‐analysis of microarray data of cells in the Leydig cell lineage

Transcriptome dataset of rat testes during the course of Leydig cell regeneration after EDS treatment was previously published.^18^ In the current study, we performed re‐analysis of the dataset for the expression of *Fgf* members.

### 2.3 Leydig cell regeneration model after EDS

Twenty‐four 60‐day‐old male Sprague Dawley rats were used and acclimated to the new animal room for a week. To deplete Leydig cells from the adult testis, each rat was intraperitoneally injected EDS (75 mg/kg of body weight). EDS was dissolved in a mixture of dimethyl sulphoxide: H_2_O (1:3, v/v) and then an aliquot of 200 µL was injected. Leydig cell‐depleted rats were randomly divided into three groups with each group of eight rats. FGF16 was dissolved in normal saline and an aliquot of 20 µL for each testis was used for intratesticular injection. Each testis daily received an injection of 0 (normal saline), 10 or 100 ng/testis FGF16, respectively, from post‐EDS day 14 for 14 days. This time‐course of administration regimen was adopted because progenitor Leydig cells begin to emerge from stem Leydig cells on post‐EDS day 14.^19^ Fourteen days after FGF16 treatment, rats were killed and drops of blood were collected. The serum samples were taken and stored at −20°C for the measurement of testosterone, LH and FSH levels. One testis per rat was frozen in −80°C for (quantitative real‐time PCR) qPCR and Western blotting analysis. The contralateral testis was fixed in Bouin's solution for immunohistochemical staining.

### 2.4 Measurement of serum and medium testosterone levels

Immulite2000 Total Testosterone kit was used to measure serum or medium testosterone concentrations as previously described.^20^ The minimal detection limit of serum testosterone was 0.2 ng/mL.

### 2.5 ELISA measurement of serum LH and FSH levels

Serum levels of LH and FSH were measured using enzyme‐linked immunosorbent assay (ELISA) kits according to the manufacturer's instructions (Chemicon, Temecula, CA, USA) as previously described.^20^ Briefly, serum sample and assay diluent were cultured in the 96‐well plate at room temperature. Then, peroxidase‐conjugated IgG anti‐LH or anti‐FSH agent was added and incubated, followed by washing steps and adding the substrate to initiate the reaction. A microplate reader was set at 550 nm with correction wavelength at 450 nm to read the data for LH or FSH.

### 2.6 Immunohistochemical staining of the testis

Immunohistochemical staining kit (Vector, Burlingame, CA, USA) was used as previously described.^20^ Eight testes per group were used and testis samples were prepared and embedded in paraffin in a tissue array block in TMA‐MASTER (3Dhistech, Budapest, Hungary). Tissue‐array samples were dehydrated in ethanol and xylene and then embedded in paraffin. Transverse sections (6 µm) were sliced and mounted on glass slides. Antigen retrieval was performed by heating for 10 minutes in 10 mmol/L (pH 6.0) of citrate buffer. Endogenous peroxidase activity was blocked by 0.5% of H_2_O_2_ in methanol for 30 minutes. Sections were then incubated with primary antibodies, CYP11A1 (a biomarker of all Leydig cells) or HSD11B1 (a biomarker for Leydig cells at the advanced stage). These polyclonal antibodies were diluted 1:200 (v/v). Antibody‐antigen complexes were checked after adding diaminobenzidine. Brown cytoplasmic staining designates a Leydig cell. The sections were counterstained with Mayer haematoxylin. The sections were then dehydrated in graded concentrations of alcohol and covered with resin (Thermo Fisher Scientific, Waltham, UK). Non‐immune rabbit IgG was used as the negative control.

### 2.7 Counting Leydig cells

To count CYP11A1‐positive and HSD11B1‐positive Leydig cells, the sampling of the testis was performed according to a fractionator technique as previously described.^20^ In brief, each testis was sliced in eight blocks, of which two blocks were randomly selected. Then, blocks were further sliced in four pieces and one piece was randomly selected from a total eight pieces. These pieces of testis were embedded in paraffin in a tissue array as above. Paraffin blocks were sectioned as above. Ten sections were randomly sampled from each testis per rat. Sections were used for immunohistochemical staining as above. Pictures were taken and total microscopic fields per section were counted. The total number of Leydig cells was calculated by multiplying the number of Leydig cells counted in a known fraction of the testis by the inverse of the sampling probability.

### 2.8 Semi‐quantitative measurement of CYP11A1 and HSD11B1 protein levels

CYP11A1 and HSD11B1 are the proteins in Leydig cells. Immunohistochemical staining of CYP11A1 and HSD11B1 in the tissue array were performed as above. The measurement of CYP11A1 and HSD11B1 protein levels was performed as previously described.^20^ Briefly, the densities of CYP11A1 or HSD11B1 and background area nearby were measured using the Image‐Pro Plus6.0 (Media Cybernetics, Silver Spring, MD, USA). The net density of CYP11A1 or HSD11B1 was calculated after subtracting the density of the background area. More than 50 Leydig cells in each testis were counted and the density of each testis was averaged as one sample size.

### 2.9 Immunofluorescent staining and calculation of proliferation of Leydig cells

Double staining of CYP11A1 (for Leydig cells) and PCNA (for proliferating cells) were performed by an immunofluorescent method as previously described.^21^ Sections were prepared in the tissue array as above. Sections were incubated with the primary antibody of PCNA for 60 minutes and then washed and incubated with the CYP11A1 antibody for double staining. Fluorescent secondary antibody (Alexa‐conjugated anti‐rabbit or anti‐mouse IgG, 1:500) were used after the primary antibodies. Sections were visualized under a fluorescent microscope (Olympus, Japan). Green colour (CYP11A1) in the cytoplasm designates a Leydig cell and red colour (PCNA) in the nucleus designates a proliferating cell.

### 2.10 Isolation and culture of seminiferous tubules

In order to investigate whether FGF16 affects the stem Leydig cell development, an in vitro culture system of stem Leydig cells on the surface of the seminiferous tubules was used as previously described.^22^ One 90‐day‐old male Sprague Dawley rat was treated with EDS (75 mg/kg body weight) as above. The rat was killed by CO_2_ on post‐EDS day 7 and Leydig cells were depleted.^22^ Testes were placed in MEM‐199 and decapsulated in the sterile condition. Seminiferous tubules were mechanically separated from the interstitial parts. The tubules were sliced into about one‐inch long fragment and distributed randomly into a 12‐well plate, with each well containing equivalent parts of tubules. Seminiferous tubules were cultured with Leydig cell differentiation medium (LDM), which contains 5 ng/mL LH, 5 mmol/L insulin‐transferrin‐selenium, and 5 mmol/L lithium chloride in DMEM: F12 medium (1:1, v/v, pH 7.2) supplemented with 0.1% bovine serum albumin, 15 mmol/L HEPES, 2.2 mg/mL sodium bicarbonate, and penicillin/streptomycin (100 U/mL and 100 mg/mL). The plate was placed in a humidified atmosphere of 5% CO_2_ at 34°C for 2 weeks. At the end of 2 weeks, the stem Leydig cells differentiate into adult Leydig cells, which are capable of secreting testosterone to the medium, which can be detected. FGF16 was added to LDM together with LDM. Medium testosterone level was measured as above.

### 2.11 EdU incorporation into stem Leydig cells

EdU incorporation into stem Leydig cells on the surface of the seminiferous tubules was measured using EdU Alexa Fluor Kit (Life Technologies, USA) as previously described.^23^ In brief, the freshly isolated seminiferous tubules as above were cultured in M199 medium and treated with 0, 1, 10 ng/mL FGF16 for 7 days. Then, 1:1000 (v/v) diluted EdU was added to the well having seminiferous tubules and EdU incorporation lasted for 24 hours. Tubules were washed with PBS, fixed in 4% paraformaldehyde, incubated with the reaction solution, and mounted on a glass slide. The EdU staining was visualized under a fluorescence microscope (Olympus, Japan) and images were captured. EdU‐positive (green colour in the nucleus) cells on the surface of the tubules were counted using the ImageProPlus 7.0 software (Media Cybernetics, Rockville, MD, USA).

### 2.12 Quantitative real‐time PCR (qPCR)

Testis and seminiferous tubule samples were homogenized in Trizol agent (Invitrogen, Carlsbad, CA, USA) for total RNA purification as previously described.^20^ After extraction, the concentration of total RNA was measured by a plate reader at OD260. The first strand of cDNA was biosynthesized and used as the template for qPCR amplification. The following Leydig cell mRNA levels, *Lhcgr*, *Scarb1*, *Star*, *Cyp11a1*, *Hsd3b1*, *Cyp17a1*, *Hsd17b3*, *Srd5a1* and *Hsd11b1*, were measured using SYBR Green qPCR Kit (Roche, Basel, Switzerland) in CFX96TM PCR equipment (Bio‐Rad, Hercules, CA, USA). The reaction mixture has 7.5 μL SYBR Green Mix, 0.75 μL forward and 0.75 μL reverse primers, 0.02 μg diluted cDNA, and 4 μL RNA‐free water. The procedure of qPCR was as the following: 95°C for 5 minutes, followed by 40 cycles of 95°C for 10 seconds and 60°C for 30 seconds. The Bio‐Rad CFX Manager Software was used to analyse the qPCR data. The specificity of the fluorescence signal was determined by both melting curve analysis and gel electrophoresis. The steady‐state levels of mRNAs were determined by a standard curve method. Ct values were collected for the standard curve and the mRNA levels were normalized to *Rps16*, the internal control. The primers and gene names were listed in Supplementary Table S2.

### 2.13 Western blot analysis

Western blotting was carried out as previously described.^20^ Briefly, the testis or seminiferous tubules were homogenized in the lysis buffer (Bocai, Shanghai, China). The protein concentrations were measured using BCA Protein Assay Kit (Takara, Japan) and bovine serum albumin was used as the standard. Protein samples were loaded and electrophoresed as well as transferred onto the nitrocellulose membrane. The membrane was blocked with 5% non‐fat milk in TBST buffer and incubated with primary antibodies: LHCGR, STAR, CYP11A1, HSD3B1, CYP17A1, HSD17B3, HSD11B1, INSL3 and NR5A1 at 4°C overnight. The membrane was washed and incubated with the HRP‐conjugated antibody (1:2000; Bioword, USA). The protein bands were visualized with Super‐Signal West Pico chemiluminescent substrate (Pierce Biotechnology, Radford, IL, USA) by BioRad Universal Hood Ⅱ equipment (Hercules, CA, USA). ACTB serves as a control. The protein levels were quantified using ImageJ software and normalized to ACTB.

### 2.14 Statistical analysis

Data were expressed as the mean ± SEM *P* < 0.05 was considered statistically significant. The difference of two groups was evaluated by unpaired Student’s *t* test when two groups were compared or by one‐way ANOVA followed by ad hoc Dunnett's multiple comparisons to compare with the control when three or more groups were compared. GraphPad version 6 software (La Jolla, CA, USA) was used.

## 3 RESULTS

### 3.1 The expression of Fgf members in the testis after EDS treatment

Our previous study demonstrated that EDS depleted all Leydig cells 7 days after treatment.^18^ We re‐analyed the transcriptome microarray data from the testes during the course of regeneration of Leydig cells to identify the differential expression of fibroblast growth factor members.^18^ As shown in Figure S1, 21 *Fgf* members (*Fgf1,* *Fgf2, Fgf3, Fgf4, Fgf5, Fgf6, Fgf7, Fgf8,* *Fgf9,* *Fgf10,* *Fgf11,* *Fgf12,* *Fgf13,* *Fgf14,* *Fgf15,* *Fgf16,* *Fgf17,* *Fgf20,* *Fgf21, Fgf22 *and *Fgf23*) of FGF family were identified and the *Fgf16* level was the highest. The expression levels of all FGF members were significantly increased on post‐EDS day 90 except *Fgf21* that was the highest on post‐EDS day 7. This suggests that *Fgf16* is expressed in rat testis.

### 3.2 FGF16 reduces testosterone levels in vivo

**Figure 1:**
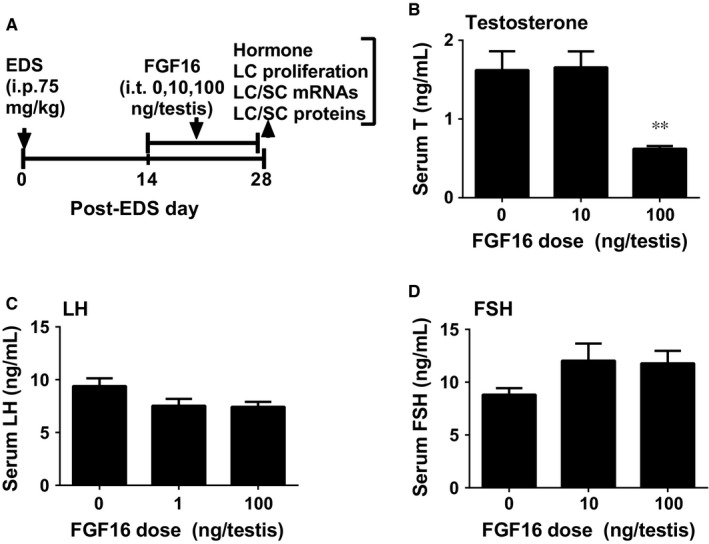
Effects of fibroblast growth factor 16 (FGF16) on serum testosterone, luteinizing hormone (LH), and follicle‐stimulating hormone (FSH) levels. A, regimen. LC, Leydig cell; SC, Sertoli cell; ip, intraperitoneal injection; it, intratesticular injection. B‐D, Serum T, LH, and FSH levels, respectively. Mean ± SEM, n = 8. Significant difference when compared to the control (FGF16 0 ng/testis) at **P < 0.01

We used a Leydig cell regeneration model to study the effect of FGF16 on rat Leydig cell development in vivo (Figure 1A). We intraperitoneally injected rats with EDS to deplete Leydig cells in the testis on post‐EDS day 7 and intratesticularly injected FGF16 (0, 10 or 100 ng/testis/day) from post‐EDS day 14 (at the stem Leydig cell stage^24^) to 28 (at the immature Leydig cell stage.^12^) We selected this regimen to examine the effects of FGF16 on the earlier stage of regeneration. Intratesticular injection of FGF16 was performed in order to avoid its systemic action. After 14‐day treatment, FGF16 did not affect rat body and testis weights when compared to the control (Table S3). FGF16 lowered serum testosterone level at a dose of 100 ng/testis (Figure 1B). However, FGF16 did not alter serum LH and FSH levels (Figure 1C and 1). These data suggest that FGF16 delays Leydig cell regeneration via direct action on the testis.

### 3.3 FGF16 increases Leydig cell number in vivo

**Figure 2:**
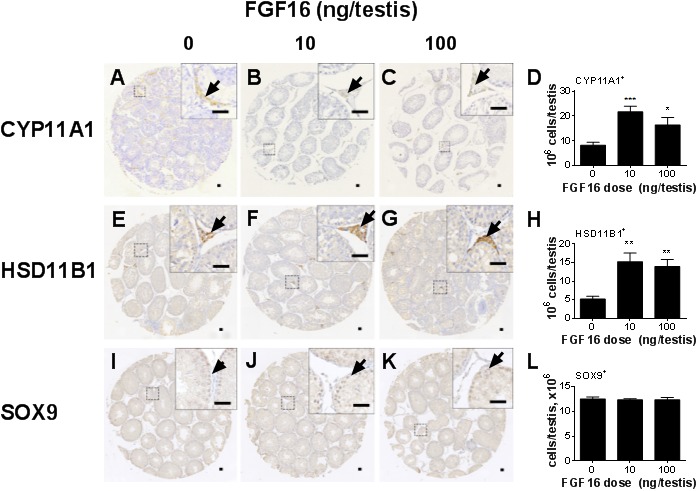
Leydig cell and Sertoli cell number after FGF16 treatment. A‐C, CYP11A1 staining for Leydig cells. E‐G, HSD11B1 staining for Leydig cells. I‐K, SOX9 staining for Sertoli cells. D, H, and L, Quantitation of cell numbers. Black arrowheads designate positive cells. Bar = 30 µm. Mean ± SEM, n = 8. Significant differences when compared to the control (0 ng/testis FGF16) at *P < 0.05, **P < 0.01, and ***P < 0.001, respectively

CYP11A1 is expressed in progenitor, immature and adult Leydig cells but not in stem Leydig cells. Morphologically, stem Leydig cells and progenitor Leydig cells are very similar and are spindle‐shaped. Therefore, stem and progenitor Leydig cells can be identified by CYP11A1 as the biomarker. Immature Leydig cells are ovoid and adult Leydig cells are round and the later are larger than the immature Leydig cells and both immature and adult Leydig cells express HSD11B1 while progenitor Leydig cells do not. Therefore, immature/adult and progenitor Leydig cells can be distinguished by HSD11B1 as the biomarker. We counted Leydig cells using a stereological method. As shown in Figure 2, FGF16 significantly increased the number of both HSD11B1‐positive and CYP11A1‐positive Leydig cells at doses of 10 and 100 ng/testis. This indicates that FGF16 stimulates stem and progenitor Leydig cell proliferation.

### 3.4 FGF16 does not alter Sertoli cell number in vivo

We counted Sertoli cells after staining these cells using SOX9 as the biomarker. As shown in Figure 2, FGF16 did not alter SOX9‐positive Sertoli cell number. As the number of Sertoli cells is stable after 21 days postpartum, the data indicate that FGF16 does not affect Sertoli cell apoptosis.

### 3.5 FGF16 increases Leydig cell mitosis in vivo

**Figure 3:**
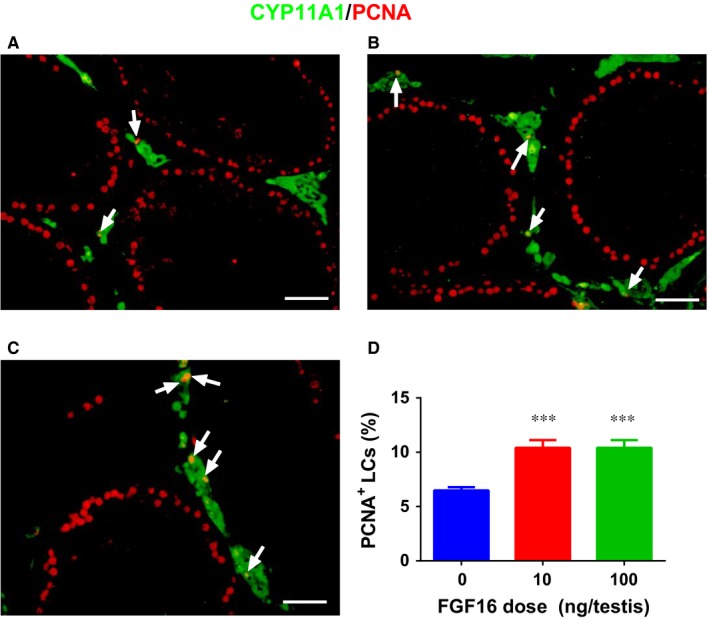
FGF16 promotes the proliferation of Leydig cells. A‐C, Images of PCNA‐labelled (red colour in the nucleus) and CYP11A1‐labelled (green colour in the cytoplasm) of cells from 0, 10 and 100 ng/testis FGF16‐treated testis, respectively. D, Quantification of the percentage of PCNA‐positive and CYP11A1‐positive cells of all Leydig cells. White arrow designates PCNA‐positive and CYP11A1‐positive Leydig cells. Bar = 50 µm. Mean ± SEM, n = 8. Significant difference when compared to the control (0 ng/testis FGF16) at ***P < 0.001

As progenitor and immature Leydig cells have the capacity of cell division, we asked whether the increased number of Leydig cells results from Leydig cell mitosis after FGF16 treatment. We labelled the mitotic cells using PCNA as the biomarker and Leydig cells using CYP11A1 as the biomarker and found that FGF16 significantly increased the percentage of PCNA‐positive Leydig cells (Figure 3). This indicates that the increase of Leydig cell numbers comes from its mitosis.

### 3.6 FGF16 down‐regulates the expression of Leydig and Sertoli cell genes in vivo

**Figure 4:**
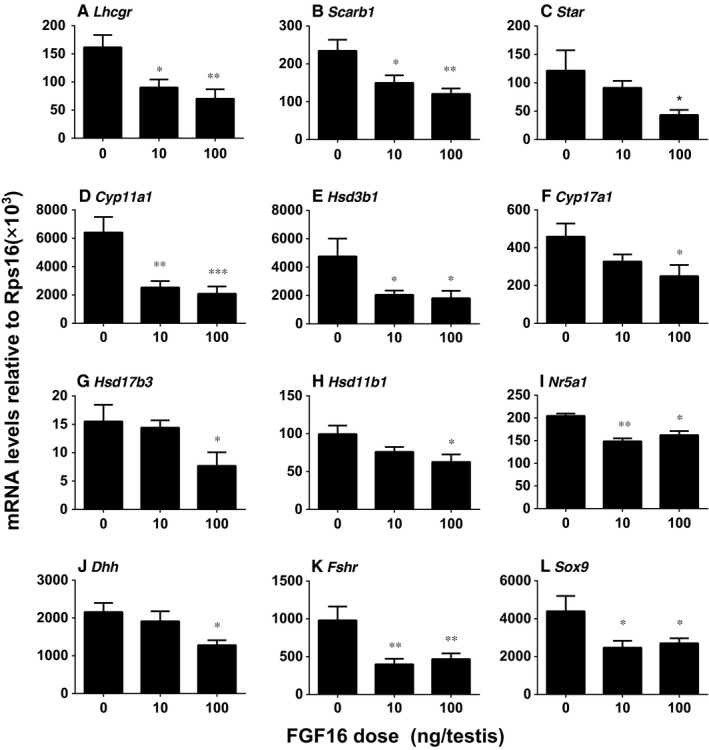
Expression levels of Leydig and Sertoli cell genes after FGF16 treatment in vivo. The mRNA levels were measured by qPCR and adjusted to the *Rps16*, the internal control. Mean ± SEM, n = 8. Significant differences when compared to the control (0 ng/testis FGF16) at *P < 0.05, **P < 0.01 and ***P < 0.001, respectively

The expression of Leydig cell genes (*Lhcgr*, *Scarb1*, *Star*, *Cyp11a1*, *Hsd3b1*, *Cyp17a1*, *Hsd17b3*, *Hsd11b1* and *Nr5a1*), which are critical for Leydig cell development and steroidogenesis and Sertoli cell genes (*Fshr*, *Dhh* and *Sox9*), which are important for Sertoli cell function, was analysed. As shown in Figure 4, FGF16 significantly down‐regulated Leydig cell gene expression at 10 and/or 100 ng/testis. This indicates that FGF16 blocks Leydig cell development. FGF16 also decreased Sertoli cell mRNA (*Fshr*, *Dhh* and *Sox9*) levels at doses of 10 and/or 100 ng/testis, indicating it also affects Sertoli cell function.

### 3.7 FGF16 affects the levels of Leydig and Sertoli cell proteins in vivo

**Figure 5:**
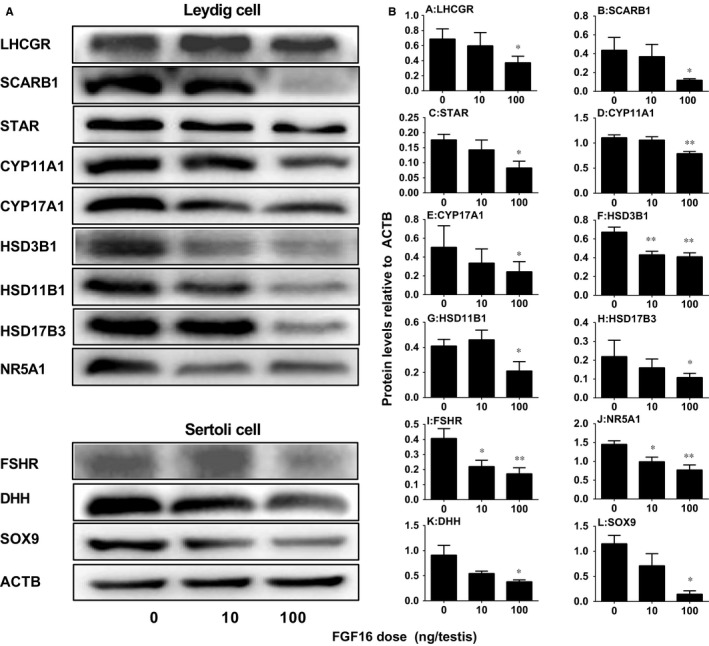
Protein levels in the testis after FGF16 treatment in vivo. Leydig cell proteins. A, Western blot bands for Leydig and Sertoli cell proteins. B, Quantification of protein levels. Mean ± SEM, n = 8. Significant differences when compared to the control (0 ng/testis FGF16) at *P < 0.05 and **P < 0.01, respectively

**Figure 6:**
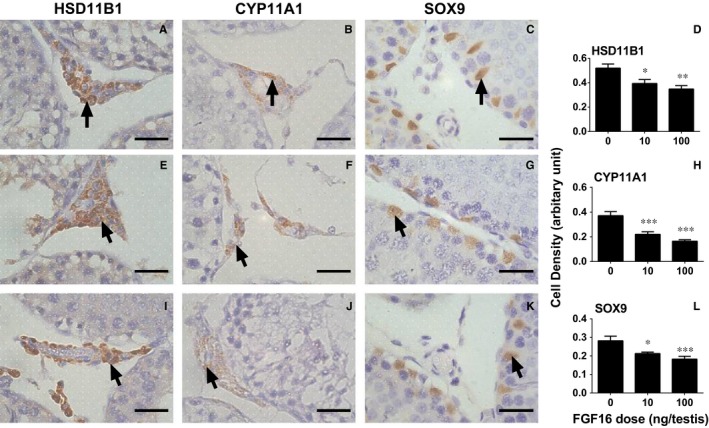
Quantitative measurement of the levels of HSD11B1, CYP11A1 and SOX9 in the cell per se after FGF16 treatment in vivo. A, E and I, HSD11B1 staining. B, F and J, CYP11A1 staining. C, G and K, SOX9 staining. D, H and K, quantitation of density. A‐C, Control; E‐G, 10 ng/testis FGF16; I‐K, 100 ng/testis FGF16. Black arrows designate positive cells. Bar =30 µm. Mean ± SEM, n = 8. Significant differences when compared to the control (0 ng/testis FGF16) at *P < 0.05, **P < 0.01 and ***P < 0.001, respectively

Western blotting was conducted to detect the protein expression levels of LHCGR, SCARB1, STAR, CYP11A1, CYP17A1, HSD3B1, HSD11B1, HSD17B3, NR5A1, FSHR, DHH, SOX9 and ACTB in the testis after FGF16 treatment (Figure 5A). Statistically, we found that FGF16 significantly lowered the levels of LHCGR, SCARB1, STAR, CYP11A1, CYP17A1, HSD3B1, HSD11B1, HSD17B3, NR5A1, FSHR, SOX9 and DHH when compared with control. We also determined the levels of CYP11A1 and HSD11B1 in Leydig cells and SOX9 in Sertoli cells per se using quantitative immunohistochemical measurement of their densities (Figure 6). Again, the levels of CYP11A1 and HSD11B1 per Leydig cell or SOX9 per Sertoli cell were significantly lower in FGF16‐treated testis. These data conform to their mRNA changes.

### 3.8 FGF16 regulates AKT1, AKT2 and ERK1/2 pathways in vivo

**Figure 7:**
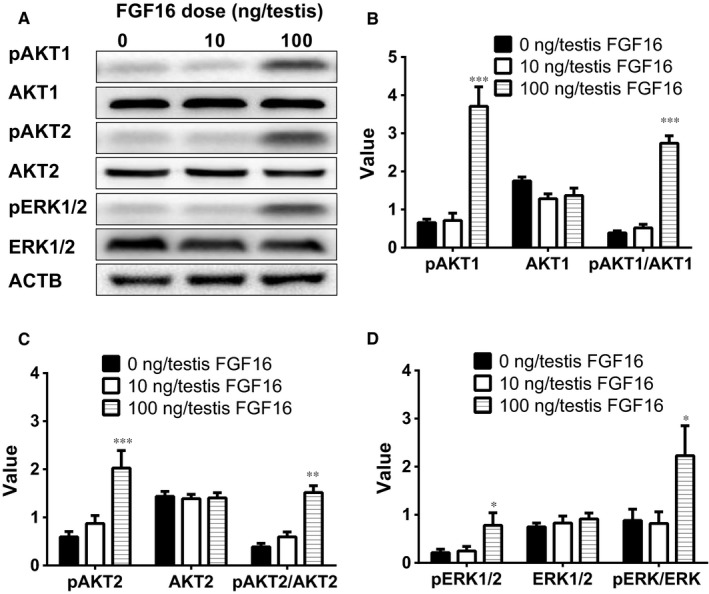
The kinase and phosphorylated kinase protein levels of the testis of rats with or without FGF16 treatment in vivo*.* A, Gel of AKT1, AKT2, ERK1/2 and their phosphorylated proteins. B‐D, Quantitative data for the levels of AKT1, AKT2, ERK1/2, and their phosphorylated proteins as well as the ratios of phosphorylated protein to each kinase. The protein levels of pAKT1, AKT1, pAKT2, AKT2, pERK1/2, ERK1/2 and ACTB (control) were analysed by Western blot in the testes from rats treated with 0, 10 and 100 ng/testis FGF16 on post‐EDS day 14 for 14 days. Mean ± SEM, n = 6‐7. Significant differences at *P < 0.05, **P <0.01, and ***P < 0.001, respectively, when compared with the control (0 ng/testis FGF16)

Many studies have shown that AKT1, AKT2 and ERK1/2 pathways participate in Leydig cell development.^25^, ^26^ We explored the down‐stream signals after FGF16 treatment in the testis by investigating AKT1, AKT2, ERK1/2, and their phosphorylation status. FGF16 significantly increased pAKT1 and pAKT2 as well as pERK1/2 levels at doses of 10 and 100 ng/testis without affecting AKT1 and AKT2 as well as ERK1/2 levels although they trended lower (Figure 7). The results indicate that FGF16 acts primarily via AKT1/AKT2 and ERK1/2 phosphorylation pathways.

### 3.9 FGF16 inhibits stem Leydig cell differentiation in vitro

**Figure 8:**
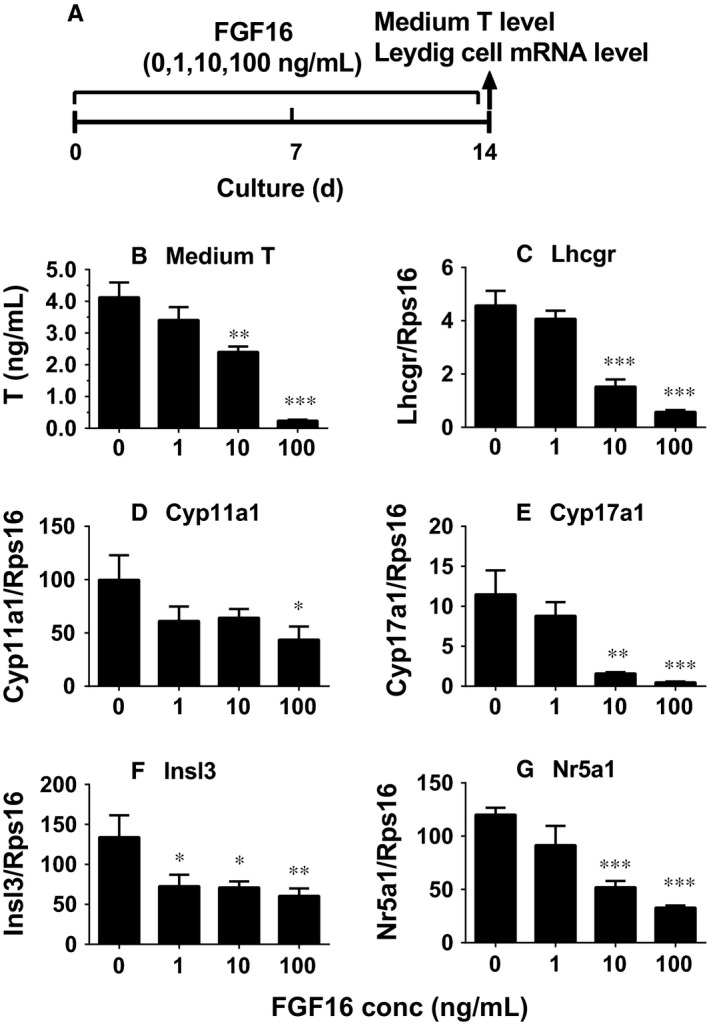
FGF16 affects medium testosterone and Leydig cell mRNA levels in vitro. EDS‐treated seminiferous tubules were cultured in LDM together with FGF16 for 14 days (A). B, Medium testosterone (T) levels. C‐G, mRNAs. Mean ± SEM, n = 6. Significant differences at *P < 0.05, **P < 0.01 and ***P < 0.001, respectively, when compared with the control (0 ng/mL FGF16)

**Figure 9:**
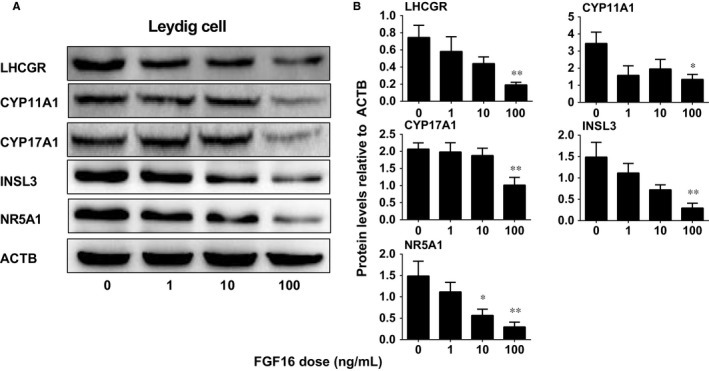
Protein levels in the seminiferous tubules after FGF16 treatment in vitro. A, Western blot images for Leydig cells. B, Quantification of protein levels. Mean ± SEM, n = 7. Significant differences when compared to the control (0 ng/mL FGF16) at *P < 0.05 and **P < 0.01, respectively

As the Leydig cell regeneration during the 28‐day period after EDS treatment covers the differentiation of stem into Leydig cells,^19^ we asked whether FGF16 affects the differentiation of stem cells into the Leydig cell lineage. We used an in vitro stem Leydig cell differentiation model.^22^ Stem Leydig cells can be induced to differentiate into Leydig cells in LDM, which secrete testosterone.^22^ Indeed, FGF16 significantly lowered testosterone levels at 10 and 100 ng/mL (Figure 8B). Then, we performed qPCR to measure the mRNA levels. Again, FGF16 significantly down‐regulated the mRNA levels of *Insl3* at 1 ng/mL, *Lhcgr*, *Cyp17a1* and *Nr5a1* at 10 and 100 ng/mL, as well as *Cyp11a1* at 100 ng/mL (Figure 8C‐G). We also measured the changes of LHCGR, CYP11A1, CYP17A1, INSL3 and NR5A1 levels and we found that they showed the similar trends to their mRNA levels (Figure 9). This indicates that FGF16 blocks the differentiation of stem Leydig cells in vitro.

### 3.10 FGF16 stimulates stem Leydig cell proliferation in vitro

**Figure 10:**
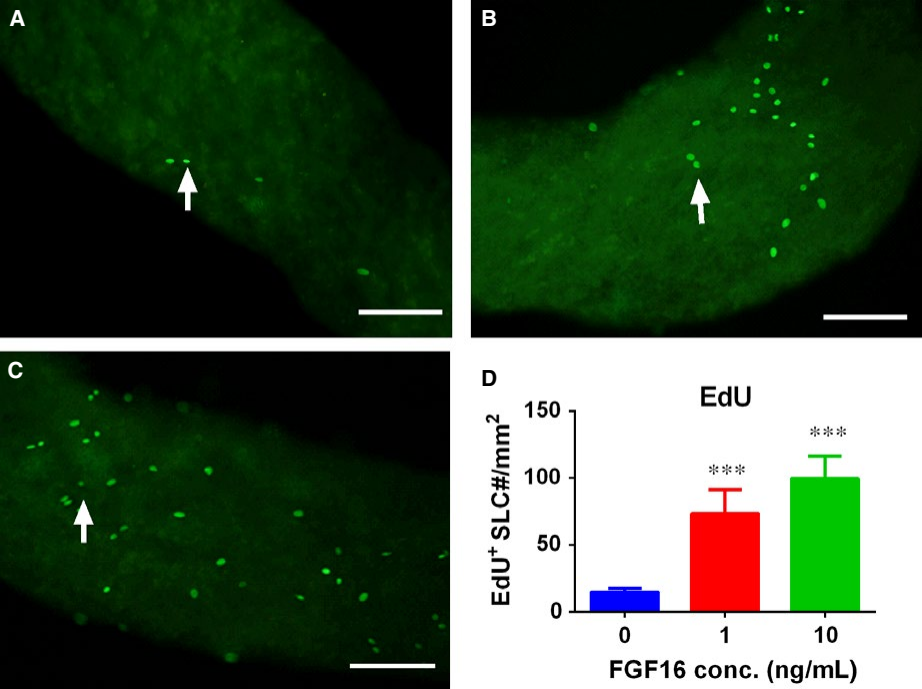
Quantitative measurement of EdU incorporation to stem Leydig cells after FGF16 treatment in vitro. A‐C, EdU staining after FGF16 treatment at 0 (control), 1 and 10 ng/mL, respectively. D, Quantitation of EdU positive stem Leydig cells (SLCs). White arrows designate EdU positive cells. Bar = 50 µm. Mean ± SEM, n = 4. Significant difference when compared to the control (0 ng/mL FGF16) at ***P < 0.001

We used EdU incorporation into proliferating cells to evaluate the proliferation of stem Leydig cells. We previously demonstrated that stem Leydig cells are attached to the surface of the seminiferous tubules and undergo mitosis.^22^ Indeed, FGF16 significantly increased EdU incorporative rate into stem Leydig cells (Figure 10), indicating that FGF16 indeed stimulates stem Leydig cell mitosis.

## 4 DISCUSSION

FGF9, one member of FGF family, has been demonstrated to play a vital role in sex determination.^28^ Herein, we identified FGF16, a member of FGF9 subfamily, which was expressed in the highest level in rat adult testis and stimulated stem and progenitor Leydig cell proliferation and blocked stem Leydig cell differentiation.

FGF9 subfamily contains three members: FGF9, FGF16 and FGF20. They do not have a classical N‐terminal signal peptide but have an internal hydrophobic sequence that functions as a non‐cleaved signal for transporting into the endoplasmic reticulum and secretion from cells.^15^ Herein, we reported that *Fgf16* was expressed in rat adult testis as the highest level (Figure S1). FGF16 has also been found to be present in the testis of the Nile tilapia, regulating its development.^29^ Leydig cells in rat testis during the development express FGF16 receptors (FGFRs), including FGFR1, FGFR2, FGFR3 and FGFR4,^30^ suggesting that FGF16 can bind these FGFRs to regulate Leydig cell development.^30^

In the EDS‐treated Leydig cell regeneration model, there is a peak of proliferative wave of interstitial cells during the first several days because of the destruction of the existing Leydig cells.^31^ Indeed, we treated EDS‐treated testis with FGF16 and discovered that FGF16 significantly increased Leydig cell mitosis as shown by the increase of PCNA‐labelling index (Figure 3) and Leydig cell number (Figure 2). We also found that FGF16 increased EdU incorporation into stem Leydig cells (Figure 10). Previous studies have proven the EdU positive cells on the seminiferous tubules were CD90 positive stem Leydig cells.^22^, ^32^ Indeed, FGF16 increased EdU incorporation to cells on the seminiferous tubules (Figures 10). This suggests that FGF16 is a mitogen for stem Leydig cells.

**Figure 11:**
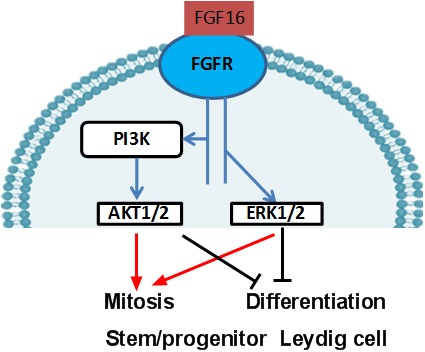
Illustration of the FGF16 signalling pathway to regulate Leydig cell development. FGF16 binds the FGFRs on the surface of stem/progenitor Leydig cells, triggering phosphorylation of PI3K, which phosphorylates AKT1 and AKT2. It also increases the ERK1/2 phosphorylation. As a result, the stem/progenitor Leydig cells undergo mitosis and exit from differentiation

In the EDS‐regenerated model, the serum testosterone levels depend on the homeostasis of Leydig cell number and steroidogenic capacity per Leydig cell. Although FGF16 significantly increased Leydig cell number (Figure 2), it remarkably down‐regulated the expression of Leydig cell specific genes and their proteins in vivo and in vitro (Figures 4, 5, 6, 8, and 9). These results suggest that the steroidogenic capacity per Leydig cell is significantly reduced after FGF16 treatment. Therefore, the serum testosterone levels were significantly lowered after 100 ng/testis FGF16 treatment (Figure 1). Although the exact mechanism that FGF16 exerts is still not well understood, the critical transcription factor, NR5A1, was down‐regulated by FGF16 (Figures 4, 5, 8, and 9). NR5A1 positively regulates the expression of many Leydig cell genes by binding their promoters.^33^, ^34^ NR5A1 is a major transcription factor for maintenance of Sertoli cell functions. Indeed, in the present study, we also found that FGF16 down‐regulated Sertoli cell gene expression as shown by the reduction of *Fshr* and *Dhh* mRNA levels (Figure 4) and their protein levels (Figure 5). *Dhh* encodes an important factor, DHH, which is secreted by Sertoli cells and it regulates Leydig cell development. Evidence has shown that in the *Dhh* knockout mice adult Leydig cells could not be well developed and only a few progenitor Leydig cells were present in the *Dhh*‐null testis.^36^

Apparently, FGF16 inhibited the differentiation of stem Leydig cells into the Leydig cell lineage in vitro as shown by the reduction of testosterone amount in the medium and the reduction in the expression levels of Leydig cell‐specific genes (Figure 8) and proteins (Figure 9).

We also asked what the down‐stream signalling FGF16 exerts. FGF16/FGFR signalling is mediated by direct recruitment of signalling molecules that bind to tyrosine autophosphorylation sites on the activated receptor and/or indirect recruitment of docking molecules that have become tyrosine phosphorylated after FGFR activation. FGF1/FGFR signal transduction activates several intracellular signalling pathways, including the phosphatidylinositol 3‐kinase (PI3K)/AKT pathways. FGF16‐FGFR signalling pathway may be mediated by down‐stream AKT1 and AKT2 as well as ERK1/2 pathways.^37^ AKT is a critical regulator of Leydig cell development. So far, three AKT isoforms, AKT1‐AKT3, were identified. AKT1 is the primary isoform in numerous mammalian tissues including testis. AKT2 is present in several insulin‐responsive tissues, regulating glucose metabolism; and AKT3 is mainly expressed in the brain to regulate brain function.^38^ Indeed, the AKT1 knockout in mice causes the testis abnormality.^39^ However, AKT2‐AKT3 double knockout in mice does not induce any abnormality of the testis,^40^ indicating that AKT1 is a major down‐stream signalling to regulate testis function. Indeed, PI3K, when it is activated, can phosphorylate AKT1.^41^ In the present study, we demonstrated that FGF16 significantly lowered the phosphorylation of AKT1 and AKT2.

The MEK‐ERK1/2 pathway is a critical signalling pathway that mediates many signals from the surface receptors of many FGFs, including FGF16.^42^ MEK phosphorylates ERK1/2, activating the down‐stream cascades. It has been reported that a Leydig cell conditional double knockout of MEK1/2, the upstream kinases of ERK1/2, induces Leydig cell hypoplasia and the decreased androgen production as well as the down‐regulation of steroidogenesis‐related genes, including *Cyp17a1*, in mice.^43^ Herein, we also observed a significant increase in phosphorylation of ERK1/2 after FGF16 treatment, indicating that this pathway may be involved in FGF16‐mediated regulation of proliferation.

In conclusion, FGF16 increases the proliferation but inhibits the differentiation of Leydig cells during their regeneration. PI3K/AKT and MEK‐ERK1/2 pathways are possibly involved in FGF16‐mediated action (11).

## CONFLICTS OF INTERESTS

The authors declared that no competing interests exist.

## Supporting information

FigS1
