# Supplementary material for: Fibroblast growth factor 16 stimulates proliferation but blocks differentiation of rat stem Leydig cells during regeneration
Source: J Cell Mol Med. 2019 Jan 22;23(4):2632–44. doi: 10.1111/jcmm.14157 (PMC6433688; doi:10.1111/jcmm.14157)
Supplement: Supplementary file 2 [file JCMM-23-2632-s002.doc]

**Supplementary Table S1 Antibody information**

| **Antibody** | **Species** | **Vendor (City, State)** | **Dilution** | |
| --- | --- | --- | --- | --- |
|  |  |  | **WB** | **HS** |
| Actin (ACTB) | Rabbit | Cell Signaling Technology (Danvers, MA) | 1:1000 | ND |
| LHCGR | Rabbit | Multi Sciences (Hangzhou, China) | 1:1000 | ND |
| SCARB1 | Rabbit | Multi Sciences (Hangzhou, China) | 1:1000 | ND |
| STAR | Rabbit | Cell Signaling Technology (Danvers, MA) | 1:1000 | ND |
| HSD3B1 | Rabbit | Multi Sciences (Hangzhou, China) | 1:500 | ND |
| CYP17A1 | Rabbit | Abcam (San Francisco, CA) | 1:1000 | ND |
| CYP11A1 | Rabbit | Cell Signaling Technology (Danvers, MA) | ND | 1::200 |
| HSD17B3 | Rabbit | Biorbyt (California, US) | ND | 1:200 |
| NR5A1 | Mouse | Santa Cruz Biotechnology (Dallas, TX) | 1:1000 | ND |
| FSHR | Rabbit | Multi Sciences (Hangzhou, China) | 1:1000 | ND |
| DHH | Mouse | Santa Cruz Biotechnology (Dallas, TX) | 1:100 | ND |
| pAKT1 | Rabbit | Abcam (San Francisco, CA) | 1:5000 | ND |
| AKT1 | Rabbit | Abcam (San Francisco, CA) | 1:2000 | ND |
| pAKT2 | Rabbit | Abcam (San Francisco, CA) | 1:500 | ND |
| AKT2 | Rabbit | Abcam (San Francisco, CA) | 1:1000 | ND |
| pERK1/2 | Mouse | Abcam (San Francisco, CA) | 1:10000 | ND |
| ERK1/2 | Mouse | Abcam (San Francisco, CA) | 1:1000 | ND |
| PCNA | Mouse | Cell Signaling Technology (Danvers, MA) | ND | 1:500 |
| INSL3 | Rabbit | Abcam (San Francisco, CA) | 1:1000 | ND |

WB = Western blot; HS = Histochemical staining; ND = Not detected.
