# Supplementary material for: Fibroblast growth factor 16 stimulates proliferation but blocks differentiation of rat stem Leydig cells during regeneration
Source: J Cell Mol Med. 2019 Jan 22;23(4):2632–44. doi: 10.1111/jcmm.14157 (PMC6433688; doi:10.1111/jcmm.14157)
Supplement: Supplementary file 3 [file JCMM-23-2632-s003.doc]

**Supplementary Table S2 Primer information**

| **Primer**  **symbol** | **Primer direction** | **Sequences (5’to 3’)** | **Product length (bp)** | **Accession** |
| --- | --- | --- | --- | --- |
| Lhcgr | Forward | CTGCGCTGTCCTGGCC | 103 | NM_012978 |
|  | Reverse | CGACCTCATTAAGTCCCCTGAA |  |  |
| Scarb1 | Forward | ATGGTACTGCCGGGCAGAT | 117 | NM_031541 |
|  | Reverse | CGAACACCCTTGATTCCTGGTA |  |  |
| Star | Forward | CCCAAATGTCAAGGAAATCA | 187 | NM_031558 |
|  | Reverse | AGGCATCTCCCCAAAGTG |  |  |
| Cyp11a1 | Forward | AAGTATCCGTGATGTGGG | 127 | NM_017286 |
|  | Reverse | TCATACAGTGTCGCCTTTTCT |  |  |
| Hsd3b1 | Forward | CCCTGCTCTACTGGCTTGC | 189 | NM_001007719 |
|  | Reverse | TCTGCTTGGCTTCCTCCC |  |  |
| Cyp17a1 | Forward | TGGCTTTCCTGGTGCACAATC | 90 | NM_012753 |
|  | Reverse | TGAAAGTTGGTGTTCGGCTGAAG |  |  |
| Hsd17b3 | Forward | TGAAAGTTGGTGTTCGGCTGAAG | 202 | NM_054007 |
|  | Reverse | TGAAAGTTGGTGTTCGGCTGAAG |  |  |
| Fshr | Forward | CCACAAGCCAATACAAACTAACT | 327 | NM_199237 |
|  | Reverse | CAAAAGTCCAGCCCAATACC |  |  |
| Dhh | Forward | AACCCCGACATAATCTTCA | 150 | NM_053367 |
|  | Reverse | CTCGTCCCAACCTTCAGT |  |  |
| Sox9 | Forward | TGCTGAACGAGAGCGAGAAG | 160 | NM_080403 |
|  | Reverse | ATGTGAGTCTGTTCGGTGGC |  |  |
| Nr5a1 | Forward | CAGAGCTGCAAAATCGACAA | 187 | NM_001191099 |
|  | Reverse | CCCGAATCTGTGCTTTCTTC |  |  |
| Hsd11b1 | Forward | TCTTCTTGGCCTACTACTAC | 91 | J05107 |
|  | Reverse | TTGCTGGCCCCTGTGACAAT |  |  |
| Insl3 | Forward | GTGGCTGGAGCAACGACA | 102 | NM_053680 |
|  | Reverse | AGAAGCCTGGTGAGGAAGC |  |  |
| Rps16 | Forward | AAGTCTTCGGACGCAAGAAA | 148 | XM_341815 |
|  | Reverse | TTGCCCAGAAGCAGAACAG |  |  |
