# Supplementary material for: Fibroblast growth factor 16 stimulates proliferation but blocks differentiation of rat stem Leydig cells during regeneration
Source: J Cell Mol Med. 2019 Jan 22;23(4):2632–44. doi: 10.1111/jcmm.14157 (PMC6433688; doi:10.1111/jcmm.14157)
Supplement: Supplementary file 4 [file JCMM-23-2632-s004.doc]

**Supplementary Table S3.** General parameters of toxicology after treatment of FGF16

| Parameters | | **Dosage (ng/testis/day)** | | |
| --- | --- | --- | --- | --- |
|  |  | **0** | **10** | **100** |
| **Body weight（g）** | |  |  |  |
|  | Post-EDS day 0 | 139.5 ± 2.806 | 138.4 ± 1.912 | 142.6 ± 1.920 |
|  | Post-EDS day 14 | 161.9 ± 6.350 | 162.5 ± 3.764 | 165.6 ± 7.766 |
|  | Post-EDS day 28 | 208.9 ± 3.524 | 194.9 ± 6.125 | 203.3 ± 4.723 |
| **Testes weight（g）** | |  |  |  |
|  | After FGF16 (14 days) | 1.510±0.0844 | 1.424±0.0941 | 1.570±0.0853 |

Mean ± SE, n = 8.
